# BAG2 structure, function and involvement in disease

**DOI:** 10.1186/s11658-016-0020-2

**Published:** 2016-09-20

**Authors:** Lixia Qin, Jifeng Guo, Qian Zheng, Hainan Zhang

**Affiliations:** 1 Department of Neurology, The Second Xiangya Hospital, Central South University, Changsha, China; 2 Department of Neurology, Xiangya Hospital, Central South University, Changsha, China

**Keywords:** BAG2, Molecular chaperones, Carcinoma, Alzheimer’s disease, Parkinson’s disease, Spinocerebellar ataxia type-3

## Abstract

Bcl2-associated athanogene 2 (BAG2) shares a similar molecular structure and function with other BAG family members. Functioning as a co-chaperone, it interacts with the ATPase domain of the heat shock protein 70 (dHsp70) through its BAG domain. It also interacts with many other molecules and regulates various cellular functions. An increasing number of studies have indicated that BAG2 is involved in the pathogenesis of various diseases, including cancers and neurodegenerative diseases. This paper is a comprehensive review of the structure, functions, and protein interactions of BAG2. We also discuss its roles in diseases, including cancer, Alzheimer’s disease, Parkinson’s disease and spinocerebellar ataxia type-3. Further research on BAG2 could lead to an understanding of the pathogenesis of these disorders or even to novel therapeutic approaches.

## Introduction

The BAG (Bcl-2-associated athanogene) family was first identified as a group of proteins that prevent cell death through their interaction with Bcl-2 [1, 2]. They share a conserved region at their C-terminal. It directly interacts through the BAG domain with the ATPase domain of Hsp70/Hsc70 molecular chaperones [3, 4]. By binding to Hsp70 at a binding site that overlaps with the Hip binding site, BAG family proteins stimulate ADP/ATP exchange and repress co-chaperone Hip binding to Hsp70, thereby dictating Hsp70–client interaction [4, 5]. In addition, BAG proteins can bind to various transcription factors and regulate a range of processes, including cell apoptosis, tumor growth, neuronal differentiation and the stress response [6, 7].

To date, six members of the family have been identified in humans: BAG1 (RAP46/HAP46), BAG2, BAG3 (CAIR-1/Bis), BAG4 (SODD), BAG5 and BAG6 (BAT3/Scythe) [4, 6]. In a study that used a yeast two-hybrid approach, BAG2 was found to be an Hsp70/Hsc70 molecular chaperone-interacting protein [5]. It shares a similar molecular structure and functions with the other members of its family, but it has some unique aspects. Recent studies have revealed that it has a potentially significant role in disease.

## BAG2 location

In humans, BAG2 is widely expressed in many tissues, including brown adipose, heart and lung tissue. BAG2 has also recently been in various types of tumor cells, including renal cell carcinoma, glioblastoma and thyroid carcinoma cells [8–11]. Immunofluorescence co-localization analysis revealed that BAG2 is associated with certain cellular components, including the mitochondria [12], endoplasmic reticulum [13, 14] and microtubules [15].

## BAG2 structure

**Fig. 1:**
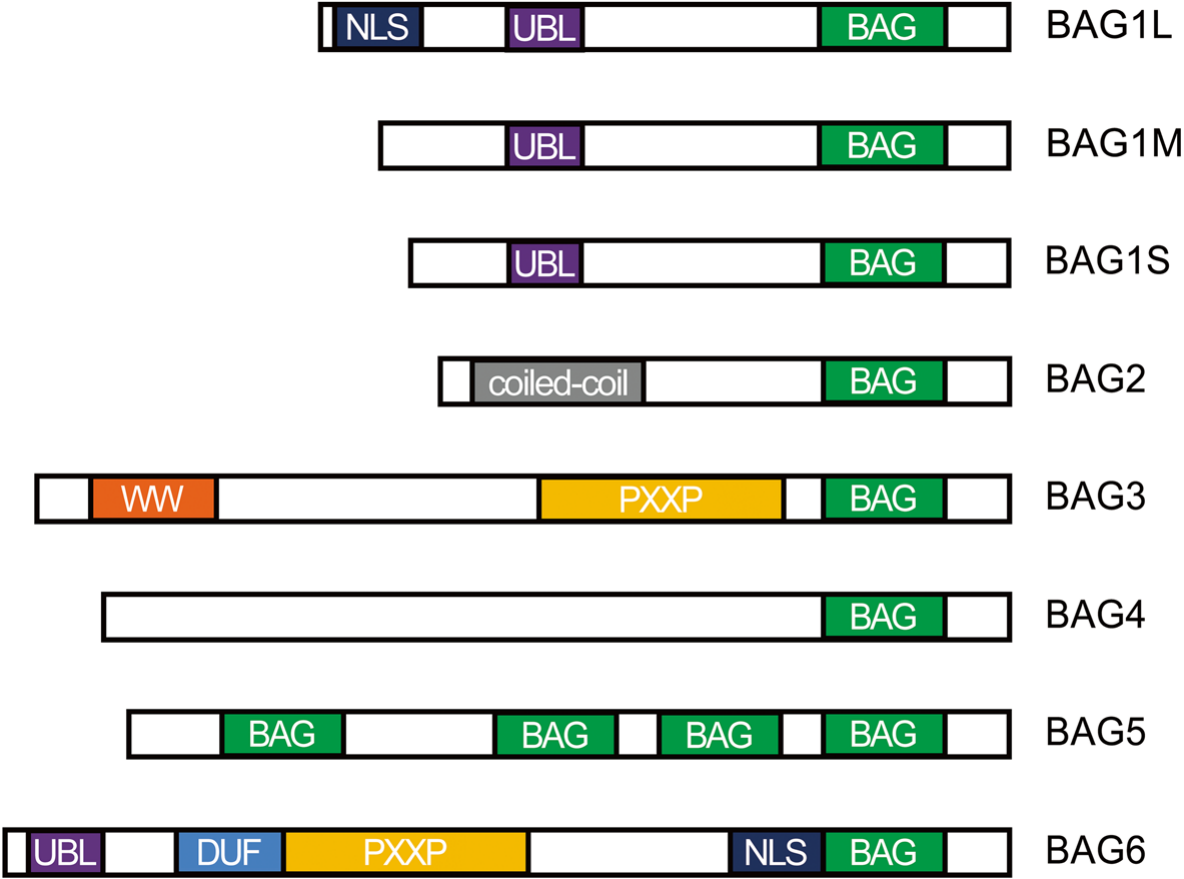
Schematic representation of the domain structure of BAG family proteins. All six reported BAG proteins contain a BAG domain at their C-terminus. The BAG2 C-terminal domain is defined as the BNB domain. There is a coiled coil domain near the amino terminus. Domains in the other BAG-family proteins include a nuclear localization signal (NLS), ubiquitin-like (UBL) domain, WW domain, DUF domain, and proline-rich regions (PXXP)

Studies on the crystal structure of BAG family proteins show that the BAG domain contains between 110 and 124 amino acids forming three anti-parallel α-helices with a relatively conserved C-terminus. Hsc70/Hsp70 binds with the second and third α-helix [16]. Additionally, there are domains at the N-terminus that mediate binding to other partners (Fig. 1).

BAG2 is an Hsp70/Hsc70 molecular chaperone-interacting protein originally discovered using yeast two-hybrid screening. It is composed of 211 amino acids. Although the C-terminus of BAG2 is classified as a BAG domain, researchers have found that it shares a low level of homology with other BAG domains. So, does the widely accepted functional model of BAG domain apply to BAG2?

Using crystallographic technology, Xu et al. [14] successfully crystallized residues 107 through 189 of murine BAG2 and determined the crystal structures of the C-terminal domain, both in the free form and in complex with the nucleotide-binding domain (NBD) of Hsp70/Hsc70. They found that the BAG2 C-terminal domain adopts a novel dimeric structure in the Hsp70/Hsc70 binding mode, and that this structure differs greatly from those of known BAG domains and from those of other Hsp70/Hsc70 nucleotide exchange factors (NEF). Moreover, they definitively showed client-binding site overlap with the Hsp70/Hsc70-binding site. They suggested that the BAG2 C-terminal domain with its atypical dimeric structure should be considered a unique Hsp70/Hsc70–NEF and proposed that it be defined as the BNB (brand new BAG) domain.

Meanwhile, crystallization and preliminary X-ray crystallographic analysis of the BAG2 N-terminal domain (NTD) suggested that it forms a dimer and adopts a folded conformation distinct from any other domains annotated in the Pfam or SMART domain databases [17].

## Functions of BAG2 regions

The BAG2 BNB domain harbors dual functions of nucleotide exchange and client binding. When binding with ATP, Hsp70 exhibits low substrate affinity, whereas in the ADP-bound state, it has high affinity for its substrates. As Hsp70-mediated protein refolding is limited by an inherently low rate of nucleotide exchange, efficient protein refolding requires interaction between Hsp70 and NEFs. Previous studies have indicated that BAG2 binds with high affinity to the ATPase domain of Hsp70 and inhibits its chaperone activity in a Hip-repressible manner. The NEF function of BAG2, which accelerates the ATPase cycle, can affect folding, aggregation and degradation reactions in different ways depending on the associated client and the cooperation with other chaperones and co-chaperones [4, 5, 14]. In addition to being an NEF, BAG2 also has intrinsic chaperone client-binding activity.

Aside from the formation of the BAG–Hsp70 complex, BAG proteins functionally interact with a variety of binding partners and coordinate diverse cellular processes, such as stress signaling, cell division, cell death and cell differentiation [5, 6, 18]. Additionally, BAG2 is an inhibitor of the Hsp70-binding E3 ubiquitin ligase CHIP (carboxyl-terminus of Hsp70-interacting protein) [19]. BAG2 inhibits CHIP-mediated ubiquitination, which requires the BAG2 NTD [20]. However, the exact mechanism by which BAG2 NTD is involved in proteasomal degradation remains to be elucidated.

### Interaction with molecular chaperones

**Fig. 2:**
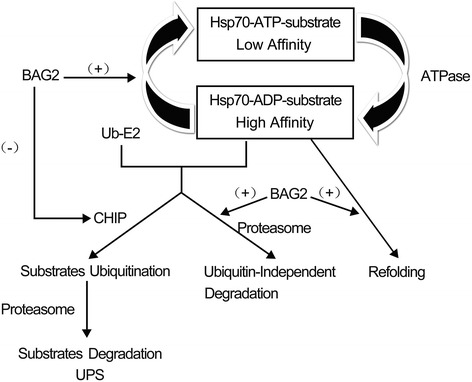
BAG2 interacts with the molecular chaperone Hsp70, which plays a prominent role in protein homeostasis. BAG2 accelerates ADP dissociation from Hsp70 and promotes Hsp70-mediated protein refolding. BAG2 is also an inhibitor of the Hsp70-binding E3 ubiquitin ligase CHIP and inhibits CHIP-mediated ubiquitination. Importantly, BAG2 inhibits tau ubiquitination, delivering phosphorylated tau to the proteasome for degradation via a ubiquitin-independent pathway. (+) indicates promoting, (−) indicates inhibiting, Ub indicates ubiquitin and UPS indicates the ubiquitin–proteasome system

Molecular chaperones are believed to play a prominent role in protein homeostasis. They regulate the balance between protein folding, translocation, assembly, disassembly, differentiation and degradation. In cells, molecular chaperones help peptides fold correctly, prevent protein misfolding and aggregation, and promote refolding of denatured polypeptides. If promoting refolding of a misfolded protein is not possible, chaperons can promote protein degradation via the ubiquitin–proteasome pathway. In eukaryotic cells, two distinctly regulated chaperone networks perform these specific functions. One includes chaperones linked to protein synthesis (CLIPS), which are functionally and physically linked to translation machinery and assist in the folding of newly translated proteins. The other is the heat shock proteins (HSPs), which can be induced by HSF and serve to protect the proteasome from stress [21–23]. The most common heat-shock protein is Hsp70/Hsc70. It is well known that BAG2 interacts with Hsp70/Hsc70 through its BNB C-terminal domain [4]. On the one hand, the BAG2/Hsp70 complex is involved the ubiquitin–proteasome degradation system through inhibiting CHIP. On the other hand, the interaction between BAG2 and Hsp70/Hsc70 modulates Hsp70/Hsc70-mediated chaperone activity [5] (Fig. 2).

### Interaction with HSP70-binding E3 ubiquitin ligase CHIP

Two mechanisms are involved in protecting cells from damage induced by misfolded proteins, including molecular chaperones and the ubiquitin–proteasome system (UPS). Molecular chaperones help proteins fold correctly, while the UPS mainly facilitates misfolded protein degradation.

CHIP is an Hsp70-associated ubiquitin ligase that participates in the UPS by ubiquitinating misfolded proteins associated with cytoplasmic chaperones. There are at least two domains in CHIP: a u-box domain, which interacts with the ubiquitin-conjugating enzyme E2, and a TPR domain, which links to Hsp70 and Hsp90 [24–26]. Previous studies addressed the BAG2 relationship to Hsp70-related ubiquitin ligase. BAG2 homologues are present in the genomes of organisms that have CHIP, but not in organisms like fungi that lack CHIP.

In vivo, BAG2 colocalizes with CHIP, especially within the endoplasmic reticulum [14]. Using binding assays, Dai et al. [20] suggested that BAG2 associates with CHIP as part of a ternary complex with Hsc70. They also indicated that BAG2 is an efficient and specific inhibitor of CHIP-dependent ubiquitin ligase activity. Further investigation proved that BAG2 NTD inhibits the ubiquitin ligase activity of CHIP by abrogating CHIP/E2 cooperation and stimulates the chaperone-assisted maturation of CFTR [14, 19]. Researchers held that this inhibitory activity is dependent on localizing to Hsp70-CHIP chaperone complexes through the BAG2 BNB–Hsp70-NBD association. Given this, additional studies on the BAG2 NTD and full-length BAG2 are necessary to better understand their mechanisms and intracellular functions.

### Interaction with other related proteins

BAG2 is phosphorylated by MAPK-activated protein (MAPKAP) kinase 2, which is known as a primary p38 MAPK substrate that mediates several p38 MAPK-dependent processes. This phosphorylation is part of a novel signaling pathway involved in the response to extracellular stresses.

MAPK cascades integrate and process various extracellular signals by phosphorylating substrates. Various stresses, endotoxins and cytokines can activate p38, whereupon it phosphorylates and regulates downstream protein kinases to modulate various intracellular processes. Using enrichment of phosphorylated proteins, fluorescent two-dimensional difference gel electrophoresis and mass spectrometric identification of proteins, Ueda et al. [27] identified BAG2 as a candidate for targets of p38 MAPK-dependent phosphorylation in response to anisomycin treatment in HeLa cells. They confirmed that MAPKAP kinase 2 is required for phosphorylation of BAG2 in vitro and in vivo. They also provided definite evidence that Ser20, which is the starting point of the coiled-coil domain near the amino terminus of BAG2, is the site of phosphorylation by MAPKAPK-2. Thus, they proposed that the p38 MAPK–MAPKAP kinase 2–BAG2 phosphorylation cascade may be a novel signaling pathway for response to extracellular stress. Hsp90 associates with BAG2 [28] and Hsp90 is required for ERK1/2 activation [29]. Previous studies showed that BAG2 overexpression reverses nicotine-induced tau phosphorylation, which is p38-dependent. This possibly occurs as a consequence of BAG2 inhibition of ERK1/2 via association with Hsp90 and/or via BAG2-mediated degradation of phosphorylated-tau as a consequence of BAG2 phosphorylation by p38/MAPKAPK2 [30].

The BAG2 coiled coil domain functions in protein–protein interactions. Further investigation is required to determine if BAG2 interaction with other unidentified proteins through the coiled coil domain is regulated by phosphorylation of BAG2 within the complex.

## The relationship of BAG2 with diseases

BAG2 is involved in numerous diseases. Most previous studies on BAG2 emphasized its important role in cancer cell metastasis [8–11]. Recently, a growing body of evidence has implicated it in the pathogenesis of neurodegenerative diseases. In this review, we summarize the currently known functions of BAG2 in cancer and neurodegenerative disorders.

### BAG2 in cancer

Previous studies demonstrated that most of the BAG family proteins play important roles in the regulation of apoptosis, cell survival and the stress response. BAG1, BAG3, BAG4 and BAG6 have anti-apoptotic activities [31–35].

Using reverse transcription-polymerase chain reaction (RT-PCR), Wang et al. [11] found that BAG2 was induced by proteasome inhibitors in cancer cell lines derived from different histology samples, including undifferentiated thyroid cancer, colon cancer, human fibrosarcoma, HeLa cervical cancer, breast cancer, ovarian cancer, pancreatic cancer, hepatic cancer, lung cancer, kidney cancer and SH-SY5Y neuroblastoma cell lines. Moreover, upregulation of BAG2 was more obvious in cells sensitive to proteasome inhibitors. As shown in their study, prevention of BAG2 upregulation, through the use of small interfering RNAs (siRNAs), inhibited the cytotoxicity induced by MG132. In the results, they suggested that BAG2 is a novel molecule induced by proteasome inhibition, which exhibits a pro-apoptotic property in response to proteasome inhibitors. However, the explicit mechanism of BAG2 gene transcriptional activation by proteasome inhibitors and how BAG2 promotes apoptosis still remain to be elucidated.

### BAG2 in Alzheimer’s disease

Tau inclusions are classic hallmarks of many neurodegenerative diseases including Alzheimer’s disease [36]. Though the exact regulatory mechanism underlying their formation is not clearly understood, tau accumulation in neurons as ubiquitinated filaments indicates a defect in tau protein triage within the tau pathway, where tau protein is assessed for refolding or degradation. In 2009, Carrettiero et al. [37] reported an elegant mechanism of tau degradation involving the co-chaperone BAG2. They explained that BAG2 exerts a prominent role in the maintenance of the neuronal cytoskeleton via proteasome-independent degradation of phosphorylated tau.

Interestingly, BAG2 also mediates cold-induced tau hyperphosphorylation [15, 38]. It is conceivable that repression of BAG2 expression or activity by cold-sensitive pathways is a causal factor in the accumulation of cytotoxic hyperphosphorylated tau via restriction of BAG2-mediated clearance of cellular phosphorylated tau [16]. Previous studies found that miR-128 is increased in the hippocampus in the Alzheimer’s disease-affected brain [39]. Carrettiero et al. found that cellular levels of BAG2 are directly under the physiological control of miR-128a [40, 41]. These findings suggest that downregulation of BAG2 by miR-128a leads to shunting of tau degradation toward the less efficient ubiquitin-dependent pathway, and the resulting decreased strength of BAG2-mediated tau degradation pathways [42] could confer risk for neurodegeneration [37].

It has been suggested that polyphenol compounds, including curcumin, (−)-epigallocatechin-3-gallate (EGCG) and resveratrol, have the potential to prevent Alzheimer’s disease pathology due to their anti-amyloidogenic, anti-oxidative and anti-inflammatory properties [43]. Recently, researchers studied the underlying molecular mechanisms by which these polyphenol compounds may protect against Alzheimer’s disease-associated tau pathology [44]. Their results showed that primary rat cortical neurons treated with polyphenols at different concentrations significantly upregulated the levels of BAG2, a potential endogenous regulator of tau. Additionally, they suggest a potential benefit conferred by the use of the polyphenol curcumin against Alzheimer’s disease-associated tau pathology, possibly through upregulating levels of the anti-tau co-chaperone BAG2.

Recent studies indicate that BAG2 expression is negatively regulated by NF-kB signaling [45] and c-Myc [46]. Inhibition of NF-kB by JSH-23, curcumin or MG132 upregulates BAG2 expression, and overexpression of BAG2 contributes to the shift of Aβ_1–42_ from neurotrophic to neurotoxic [44]. Further research on the NF-kB–BAG2–Aβ axis may offer novel insight into the pathogenesis of Alzheimer’s disease.

### BAG2 in Parkinson’s disease

Parkinson’s disease, a progressive neurodegenerative disorder, is characterized by progressive degeneration of dopaminergic neurons and the formation of Lewy bodies, mainly in the substantia nigra pars compacta (SNpc) [47, 48]. With increasing awareness of the importance of genetic factors involved in Parkinson’s disease, attention has focused on the role of several identified genes that lead to a hereditary parkinsonian disorder.

*PINK1* is the second most common pathogenic gene associated with autosomal recessive, early onset Parkinson’s disease [49]. Previous work revealed that the interaction of PINK1 with other proteins, including Parkin [50], DJ-1 [51], Cdc37/Hsp90 complex [52], HtrA2 [53], TRAP1 [54] and BAG5 [55] might contribute to the pathogenesis of the disease. These data prompt the question of whether BAG2 also interacts with PINK1, a Parkinson’s disease-related protein. Through co-immunoprecipitation and GST pull down assays, Che et al. [56] revealed that BAG2 directly binds and stabilizes both wild-type PINK1 and the R492X PINK1 mutation by decreasing their ubiquitination. They identified a direct interaction between BAG2 and PINK1, and showed that BAG2 regulates the level of PINK1 via the UPS.

Studies in *Drosophila* models of Parkinson’s disease demonstrated that Parkin and PINK1 function in a common pathway that regulates mitochondrial quality control [57, 58]. Further investigation found that stabilization of PINK1 by BAG2 triggers Parkin-mediated mitophagy and protects neurons against 1-methyl-4-phenylpyridinium-induced oxidative stress in an in vitro cell model of Parkinson’s disease. Collectively, these findings indicate that BAG2 is an upstream regulator of the PINK1/PARKIN signaling pathway, suggesting that BAG2 may play an important role in the pathogenesis of PD [59].

BAG5 was identified as a gene that is upregulated in dopaminergic neurons following medial forebrain bundle transfection. BAG5 was also found to function as a pro-apoptotic factor enhancing dopaminergic neuron degeneration via suppression of the E3 ligase activity of parkin [60, 61]. It is unclear whether other members of the BAG family possess similar functions and therefore the relationship of BAG2 and dopaminergic neuron degeneration is still poorly understood.

### BAG2 in spinocerebellar ataxia type-3

Spinocerebellar ataxia type-3 (SCA3) is the most common dominantly inherited ataxia in the world. It is characterized by a pathological, unstable cytosine–adenine–guanine trinucleotide repeat expansion in the translated region of *ATXN3* [62, 63]. The translated polyglutamine tract located at ataxin3 is aggregated in degenerated neurons, leading to the dysfunction and degeneration of specific neuronal subpopulations [64]. In the study of upstream proteins in the UPS of ataxin3-80Q, a kind of pathogenic protein of SCA3, researchers found that BAG2 co-localizes with ataxin3-80Q in vivo and prevents the degradation of ataxin3-80Q via the UPS. Therefore, they suggested that BAG2 is the upstream protein of pathogenic ataxin3–80Q, which proved that it has important roles in neurodegenerative diseases [65].

## Conclusions and future perspectives

**Fig. 3:**
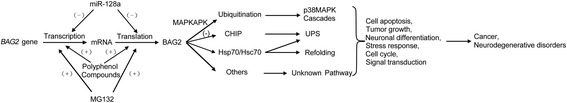
The relationship between BAG2 and disease. *BAG2* gene expression is regulated by various biochemical factors at the level of transcription and translation. BAG2 interacts with other proteins to regulate diverse biological and pathological processes, such as cell apoptosis, tumor growth, neuronal differentiation, stress response, cell cycle and signal transduction. Therefore, BAG2 may play an important role in the pathogenesis of diseases, including cancer and neurodegenerative disorders. (+) indicates promoting, (−) indicates inhibiting and UPS indicates the ubiquitin–proteasome system

Overall, BAG2 shares similar molecular structure and function with other BAG family members, but it possesses some unique features. It interacts with several proteins that are involved in many molecular pathways regulating diverse biological and pathological processes (Fig. 3). Though many molecular details of these processes remain to be discovered, previous research proved that BAG2 interacts with Hsp70/Hsc70, which further modulates Hsp70-mediated chaperone activity, and inhibits CHIP-dependent ubiquitin ligase activity. Moreover, BAG2 has been identified as a primary substrate of p38 MAPK, mediating several p38 MAPK-dependent processes.

A number of studies of tumor cell lines of different origin have shown that BAG2 influences cell survival and exhibits a pro-apoptotic property. More importantly, recent findings suggest that BAG2 takes part in the aggregation of aberrant proteins through chaperone-mediated refolding and ubiquitin–proteasome degradation pathways. It has potentially significant roles in neurodegenerative disease pathogenesis. For example, BAG2 delivers tau to the proteasome for ubiquitin-independent degradation, and stabilizes both PINK1 and pathogenic ataxin3-80Q by decreasing its ubiquitination. Further research on BAG2-related molecular pathways may give additional insight into the pathogenesis of numerous diseases and help to devise future strategies for diagnosis and treatment.

## Abbreviations

BAG: Bcl2-associated athanogene
BNB: Brand new BAG
CHIP: Carboxyl-terminus of Hsp70-interacting protein
CLIPS: Chaperones linked to protein synthesis
EGCG: (−)-epigallocatechin-3-gallate
HSPs: Heat shock proteins
MAPKAP: MAPK-activated protein
NBD: Nucleotide-binding domain
NEF: Nucleotide exchange factor
NTD: N-terminal domain
SCA3: Spinocerebellar ataxia type-3
siRNAs: small interfering RNAs
UPS: Ubiquitin–proteasome system

## References

[1] Takayama S, Sato T, Krajewski S, Kochel K, Irie S, Millan JA (1995). Cloning and functional analysis of BAG-1: a novel Bcl-2-binding protein with anti-cell death activity. Cell.

[2] Lee JH, Takahashi T, Yasuhara N, Inazawa J, Kamada S, Tsujimoto Y (1999). Bis, a Bcl-2-binding protein that synergizes with Bcl-2 in preventing cell death. Oncogene.

[3] Brive L, Takayama S, Briknarová K, Homma S, Ishida SK, Reed JC (2001). The carboxyl-terminal lobe of Hsc70 ATPase domain is sufficient for binding to BAG1. Biochem Biophys Res Commun.

[4] Kabbage M, Dickman MB (2008). The BAG proteins: a ubiquitous family of chaperone regulators. Cell Mol Life Sci.

[5] Takayama S, Xie Z, Reed JC (1999). An evolutionarily conserved family of Hsp70/Hsc70 molecular chaperone regulators. J Biol Chem.

[6] Takayama S, Reed JC (2001). Molecular chaperone targeting and regulation by BAG family proteins. Nat Cell Biol.

[7] Doong H, Vrailas A, Kohn EC (2002). What’s in the ‘BAG’? A functional domain analysis of the BAG-family proteins. Cancer Lett.

[8] Slater AA, Alokail M, Gentle D, Yao M, Kovacs G, Maher ER (2013). DNA methylation profiling distinguishes histological subtypes of renal cell carcinoma. Epigenetics.

[9] Klopflesch R, Meyer A, Lenze D, Hummel M, Gruber AD (2013). Canine cutaneous peripheral nerve sheath tumours versus fibrosarcomas can be differentiated by neuroectodermal marker genes in their transcriptome. J Comp Pathol.

[10] Asperger A, Renner C, Menzel M, Gebhardt R, Meixensberger J, Gaunitz F (2011). Identification of factors involved in the anti-tumor activity of carnosine on glioblastomas using a proteomics approach. Cancer Invest.

[11] Wang HQ, Zhang HY, Hao FJ, Meng X, Guan Y, Du ZX (2008). Induction of BAG2 protein during proteasome inhibitor-induced apoptosis in thyroid carcinoma cells. Br J Pharmacol.

[12] Li S, Banck M, Mujtaba S, Zhou MM, Sugrue MM, Walsh MJ (2010). p53-induced growth arrest is regulated by the mitochondrial SirT3 deacetylase. PLoS One.

[13] Saxena A, Banasavadi-Siddegowda YK, Fan Y, Bhattacharya S, Roy G, Giovannucci DR, Frizzell RA (2012). Human heat shock protein 105/110 kDa (Hsp105/110) regulates biogenesis and quality control of misfolded cystic fibrosis transmembrane conductance regulator at multiple levels. J Biol Chem.

[14] Xu Z, Page RC, Gomes MM, Kohli E, Nix JC, Herr AB (2008). Structural basis of nucleotide exchange and client binding by the Hsp70 cochaperone Bag2. Nat Struct Mol Biol.

[15] de Paula CA, Santiago FE, de Oliveira AS, Oliveira FA, Almeida MC, Carrettiero DC (2015). The co-chaperone BAG2 mediates cold-induced accumulation of phosphorylated tau in SH-SY5Y Cells. Cell Mol Neurobiol.

[16] Sondermann H, Scheufler C, Schneider C, Hohfeld J, Hartl FU, Moarefi I (2001). Structure of a Bag/Hsc70 complex: convergent functional evolution of Hsp70 nucleotide exchange factors. Science.

[17] Page RC, Xu Z, Amick J, Nix JC, Misra S (2012). Crystallization and preliminary X-ray crystallographic analysis of the Bag2 amino-terminal domain from Mus musculus. Acta Crystallogr Sect F: Struct Biol Cryst Commun.

[18] Takayama S, Bimston DN, Matsuzawa S, Freeman BC, Aime-Sempe C, Xie Z (1997). BAG-1 modulates the chaperone activity of Hsp70Hsc70. EMBO J.

[19] Arndt V, Daniel C, Nastainczyk W, Alberti S, Höhfeld J (2005). BAG-2 acts as an inhibitor of the chaperone-associated ubiquitin ligase CHIP. Mol Biol Cell.

[20] Dai Q, Qian SB, Li HH, McDonough H, Borchers C, Huang D (2005). Regulation of the cytoplasmic quality control protein degradation pathway by BAG2. J Bio Chemi.

[21] Albanèse V, Yam AY, Baughman J, Parnot C, Frydman J (2006). Systems analyses reveal two chaperone networks with distinct functions in eukaryotic cells. Cell.

[22] Haslbeck M, Franzmann T, Weinfurtner D, Buchner J (2005). Some like it hot: the structure and function of small heat-shock proteins. Nat Struct Mol Biol.

[23] Chen B, Retzlaff M, Roos T, Frydman J (2011). Cellular strategies of protein quality control. Cold Spring Harb Perspect Biol.

[24] Jiang J, Ballinger CA, Wu Y, Dai Q, Cyr DM, Höhfeld J (2001). CHIP is a U-box-dependent E3 ubiquitin ligase: identification of Hsc70 as a target for ubiquitylation. J Biol Chem.

[25] Ballinger CA, Connell P, Wu Y, Hu Z, Thompson LJ, Yin LY (1999). Identification of CHIP, a novel tetratricopeptide repeat-containing protein that interacts with heat shock proteins and negatively regulates chaperone functions. Mol Cell Biol.

[26] Connell P, Ballinger CA, Jiang J, Wu Y, Thompson LJ, Höhfeld J (2001). The co-chaperone CHIP regulates protein triage decisions mediated by heat-shock proteins. Nat Cell Biol.

[27] Ueda K, Kosako H, Fukui Y, Hattori S (2004). Proteomic identification of Bcl2-associated athanogene 2 as a novel MAPK-activated protein kinase 2 substrate. J Biol Chem.

[28] Gano JJ, Simon JA (2010). A proteomic investigation of ligand-dependent HSP90 complexes reveals CHORDC1 as a novel ADP-dependent HSP90-interacting protein. Mol Cell Proteomics.

[29] Sétáló G, Singh M, Guan X, Toran-Allerand CD (2002). Estradiol-induced phosphorylation of ERK1/2 in explants of the mouse cerebral cortex: the roles of heatshock protein 90 (Hsp90) and MEK2. J Neurobiol.

[30] de Oliveira AS, Santiago FE, Balioni LF, Ferrari Mde F, Almeida M, Carrettiero DC (2016). BAG2 expression dictates a functional intracellular switch between the p38-dependent effects of nicotine on tau phosphorylation levels via the α7 nicotinic receptor. Exp Neurol.

[31] Kudoh M, Knee DA, Takayama S, Reed JC (2002). Bag1 proteins regulate growth and survival of ZR-75-1 human breast cancer cells. Cancer Res.

[32] Romano MF, Festa M, Pagliuca G, Lerose R, Bisogni R, Chiurazzi F (2003). BAG3 protein controls B-chronic lymphocytic leukaemia cell apoptosis. Cell Death Differ.

[33] Bruchmann A, Roller C, Walther TV, Schäfer G, Lehmusvaara S, Visakorpi T (2013). Bcl-2 associated athanogene 5 (Bag5) is overexpressed in prostate cancer and inhibits ER-stress induced apoptosis. BMC Cancer.

[34] Tschopp J, Martinon F, Hofmann K (1999). Apoptosis: Silencing the death receptors. Curr Biol.

[35] Binici J, Koch J (2014). BAG-6, a jack of all trades in health and disease. Cell Mol Life Sci.

[36] Fontaine SN, Sabbagh JJ, Baker J, Martinez-Licha CR, Darling A, Dickey CA (2015). Cellular factors modulating the mechanism of tau protein aggregation. Cell Mol Life Sci.

[37] Carrettiero DC, Hernandez I, Neveu P, Papagiannakopoulos T, Kosik KS (2009). The cochaperone BAG2 sweeps paired helical filament- insoluble tau from the microtubule. J Neurosci.

[38] Carrettiero DC, Santiago FE, Motzko-Soares ACP, Almeida MC (2016). Temperature and toxic Tau in Alzheimer’s disease: new insights. Temperature.

[39] Lukiw WJ (2007). Micro-RNA speciation in fetal, adult and Alzheimer’s disease hippocampus. Neuroreport.

[40] Krek A, Grün D, Poy MN, Wolf R, Rosenberg L, Epstein EJ (2005). Combinatorial microRNA target predictions. Nat Genet.

[41] Grimson A, Farh KK, Johnston WK, Garrett-Engele P, Lim LP, Bartel DP (2007). MicroRNA targeting specificity in mammals: determinants beyond seed pairing. Mol Cell.

[42] Dickey CA, Yue M, Lin WL, Dickson DW, Dunmore JH, Lee WC (2006). Deletion of the ubiquitin ligase CHIP leads to the accumulation, but not the aggregation, of both endogenous phospho- and caspase-3-cleaved tau species. J Neurosci.

[43] Kim J, Lee HJ, Lee KW (2010). Naturally occurring phytochemicals for the prevention of Alzheimer’s disease. J Neurochem.

[44] Patil SP, Tran N, Geekiyanage H, Liu L, Chan C (2013). Curcumin-induced upregulation of the anti-tau cochaperone BAG2 in primary rat cortical neurons. Neurosci Lett.

[45] Santiago FE, Almeida MC, Carrettiero DC (2015). BAG2 is repressed by NF-kB signaling, and its overexpression is sufficient to shift Aβ1-42 from neurotrophic to neurotoxic in undifferentiated SH-SY5Y neuroblastoma. J Mol Neurosci.

[46] Zhang J, Lou X, Yang S, He S, Yang L, Liu M (2012). BAG2 is a target of the c-Myc gene and is involved in cellular senescence via the p21(CIP1) pathway. Cancer Lett.

[47] Halliday GM, McCann H (2010). The progression of pathology in Parkinson’s disease. Ann N Y Acad Sci.

[48] Halliday G, Hely M, Reid W, Morris J (2008). The progression of pathology in longitudinally followed patients with Parkinson’s disease. Acta Neuropathol.

[49] Bonifati V, Rohé CF, Breedveld GJ, Fabrizio E, De Mari M, Tassorelli C (2005). Early-onset parkinsonism associated with PINK1 mutations: frequency, genotypes, and phenotypes. Neurology.

[50] Park J, Lee SB, Lee S, Kim Y, Song S, Kim S (2006). Mitochondrial dysfunction in Drosophila PINK1 mutants is complemented by parkin. Nature.

[51] Tang B, Xiong H, Sun P, Zhang Y, Wang D, Hu Z (2006). Association of PINK1 and DJ-1 confers digenic inheritance of early-onset Parkinson’s disease. Hum Mol Genet.

[52] Weihofen A, Ostaszewski B, Minami Y, Selkoe DJ (2008). Pink1 Parkinson mutations, the Cdc37/Hsp90 chaperones and Parkin all influence the maturation or subcellular distribution of Pink1. Hum Mol Genet.

[53] Plun-Favreau H, Klupsch K, Moisoi N, Gandhi S, Kjaer S, Frith D (2007). The mitochondrial protease HtrA2 is regulated by Parkinson’s disease-associated kinase PINK1. Nat Cell Biol.

[54] Pridgeon JW, Olzmann JA, Chin LS, Li L (2007). PINK1 protects against oxidative stress by phosphorylating mitochondrial chaperone TRAP1. PLoS Biol.

[55] Wang X, Guo J, Fei E, Mu Y, He S, Che X (2014). BAG5 protects against mitochondrial oxidative damage through regulating PINK1 degradation. PLoS One.

[56] Che X, Tang B, Wang X, Chen D, Yan X, Jiang H (2013). The BAG2 protein stabilises PINK1 by decreasing its ubiquitination. Biochem Biophys Res Commun.

[57] Park J, Lee SB, Lee S, Kim Y, Song S, Kim S (2006). Mitochondrial dysfunction in Drosophila PINK1 mutants is complemented by parkin. Nature.

[58] Clark IE, Dodson MW, Jiang C, Cao JH, Huh JR, Seol JH (2006). Drosophila pink1 is required for mitochondrial function and interacts genetically with parkin. Nature.

[59] Qu D, Hage A, Don-Carolis K, Huang E, Joselin A, Safarpour F (2015). BAG2 gene-mediated regulation of PINK1 protein is critical for mitochondrial translocation of PARKIN and neuronal survival. J Biol Chem.

[60] Kalia SK, Lee S, Smith PD, Liu L, Crocker SJ, Thorarinsdottir TE (2004). BAG5 inhibits parkin and enhances dopaminergic neuron degeneration. Neuron.

[61] Chung KK, Dawson TM (2004). Parkin and Hsp70 sacked by BAG5. Neuron.

[62] Paulson H (2012). Machado-Joseph disease/spinocerebellar ataxia type 3. Handb Clin Neurol.

[63] Soong B, Cheng C, Liu R, Shan D (1997). Machado-Joseph disease: clinical, molecular, and metabolic characterization in Chinese kindreds. Ann Neurol.

[64] Fan HC, Ho LI, Chi CS, Chen SJ, Peng GS, Chan TM (2014). Polyglutamine (PolyQ) diseases: genetics to treatments. Cell Transplant.

[65] Che XQ, Tang BS, Wang HF, Yan XX, Jiang H, Shen L (2015). The BAG2 and BAG5 proteins inhibit the ubiquitination of pathogenic ataxin3-80Q. Int J Neurosci.

